# Prestimulus feedback connectivity biases the content of visual experiences

**DOI:** 10.1073/pnas.1817317116

**Published:** 2019-07-22

**Authors:** Elie Rassi, Andreas Wutz, Nadia Müller-Voggel, Nathan Weisz

**Affiliations:** ^a^Centre for Cognitive Neuroscience, University of Salzburg, 5020 Salzburg, Austria;; ^b^The Picower Institute for Learning & Memory, Massachusetts Institute of Technology, Cambridge, MA 02139;; ^c^Department of Brain & Cognitive Sciences, Massachusetts Institute of Technology, Cambridge, MA 02139;; ^d^Center for Biomagnetismus, Department of Neurosurgery, University Hospital, 91054 Erlangen, Germany;; ^e^Center for Mind/Brain Sciences (CIMeC), University of Trento, 38123 Trento, Italy

**Keywords:** prestimulus, connectivity, MEG, visual object perception, oscillations

## Abstract

Ongoing fluctuations in neural excitability and in networkwide activity patterns before stimulus onset have been proposed to underlie variability in near-threshold stimulus detection paradigms—that is, whether or not an object is perceived. Here, we investigated the impact of prestimulus neural fluctuations on the content of perception—that is, whether one or another object is perceived. We recorded neural activity with magnetoencephalography (MEG) before and while participants briefly viewed an ambiguous image, the Rubin face/vase illusion, and required them to report their perceived interpretation in each trial. Using multivariate pattern analysis, we showed robust decoding of the perceptual report during the poststimulus period. Applying source localization to the classifier weights suggested early recruitment of primary visual cortex (V1) and ∼160-ms recruitment of the category-sensitive fusiform face area (FFA). These poststimulus effects were accompanied by stronger oscillatory power in the gamma frequency band for face vs. vase reports. In prestimulus intervals, we found no differences in oscillatory power between face vs. vase reports in V1 or in FFA, indicating similar levels of neural excitability. Despite this, we found stronger connectivity between V1 and FFA before face reports for low-frequency oscillations. Specifically, the strength of prestimulus feedback connectivity (i.e., Granger causality) from FFA to V1 predicted not only the category of the upcoming percept but also the strength of poststimulus neural activity associated with the percept. Our work shows that prestimulus network states can help shape future processing in category-sensitive brain regions and in this way bias the content of visual experiences.

## Significance

Ongoing neural activity influences stimulus detection—that is, whether or not an object is seen. Here, we uncover how it could influence the content of what is seen. In ambiguous situations, for instance, ongoing neural fluctuations might bias perception toward one or the other interpretation. Indeed, we show increased information flow from category-selective brain regions (here, the fusiform face area [FFA]) to the primary visual cortex before participants subsequently report seeing faces rather than a vase in the Rubin face/vase illusion. Our results identify a neural connectivity pathway that biases future perception and helps determine mental content.

Ongoing fluctuations in neural activity interact with perceptual and cognitive processes. They help explain why repetitions of the same physical stimuli elicit different percepts and responses from trial to trial in both animals (1) and humans (2–5). Both local excitability changes in task-relevant sensory regions (6, 7) and neural network connectivity patterns have been shown to underlie trial-by-trial fluctuations in perception (8–11).

The paradigms to study the impact of ongoing neural activity on perception typically involve near-threshold detection and discrimination tasks, in which prestimulus neural fluctuations influence the perceptual fate of stimuli—for example, whether an object is seen (“Hit”) or not (“Miss”; e.g., refs. 8 and 10–19). Beyond mere stimulus detection and discrimination, one of the visual system’s essential functions is to identify and categorize objects and, in this way, construct the content of visual experiences (19–21). Neural correlates of object perception and categorization have been shown to rely on the information flow between the occipital and inferior temporal cortical regions (22–24). Here, we focus on the impact of neural excitability and connectivity patterns before stimulus onset on the content of perceptual operations.

Bistable perception paradigms are uniquely suited to address this question (25). In such paradigms, the brain is conflicted between multiple possible interpretations of visual content. Typical examples include the Rubin face/vase stimulus (26), the Necker cube (27), and binocular rivalry (ref. 28, as cited in ref. 29). Recent evidence from fMRI studies has shown that rivalry between two competing percepts is resolved relatively early in the visual hierarchy (e.g., refs. 30 and 31), such as in category-sensitive inferior temporal lobe regions (ref. 32, but see refs. 33 and 34 for fMRI and electrophysiological evidence showing an influence of parietal and frontal cortices; also see ref. 35 for a recent review). In particular for the Rubin face/vase illusion, greater blood-oxygen-level–dependent (BOLD) activity has been observed in the fusiform face area (FFA) when participants reported seeing faces rather than a vase (36). Importantly, this BOLD increase in FFA was also observed before stimulus onset (37), possibly because the prestimulus brain state biased perception toward the “face interpretation.” Still, a more comprehensive, mechanistic account requires means to simultaneously measure neural activity in multiple cortical areas with high temporal resolution to map out the cortical hubs and their interareal information flow before and during perception of an ambiguous stimulus. For example, enhanced BOLD activity in FFA could be a consequence either of increased feedforward activity from earlier visual regions or of increased feedback activity to earlier visual regions.

In the current study, we used a similar Rubin face/vase paradigm as in the aforementioned fMRI study (37). Advancing on previous work, we thoroughly characterized neural activity and connectivity patterns with high temporal resolution before and during perception of the ambiguous Rubin stimulus by means of magnetoencephalography (MEG). We hypothesized that a category-sensitive processing region (here, FFA) should exhibit differential prestimulus connectivity patterns preceding subsequent face vs. vase reports. Based on the “Windows to Consciousness” framework (11, 38), fluctuating connectivity levels of sensory regions shape upcoming stimulus processing (i.e., whether a stimulus is perceived or not). We extended these predictions to visual object perception and investigated whether categorical responses to the content of the Rubin stimulus were biased by local excitability, feedforward connectivity, or feedback connectivity between the primary visual cortex and the FFA.

## Results

Twenty participants took part in the MEG experiment. We showed them the Rubin face/vase stimulus briefly and asked them to report whether they had seen faces or a vase in each trial (*SI Appendix*, *Materials and Methods* for details). Vase and face reports were equally likely [Face mean: 49.9%; SD: 12.47%; range: 22.6–84.8% (*SI Appendix*, Fig. S1); *t* test against chance (50%) *t*(19) = 0.04; *P* = 0.97]. To ascertain that the reported perception was stochastic trial by trial, we analyzed the sequences of reported percepts by binning the trials into a range of 0–10 repetitions. A binomial distribution accounted well for the binned data for both vase and face trials (goodness-of-fit: *R*^2^ = 0.96 for face, *R*^2^ = 0.98 for vase) indicative of no systematic reporting of either percept. That is, during each trial a participant was equally likely to report faces or vase irrespective of the previous trial.

**Fig. 1.**
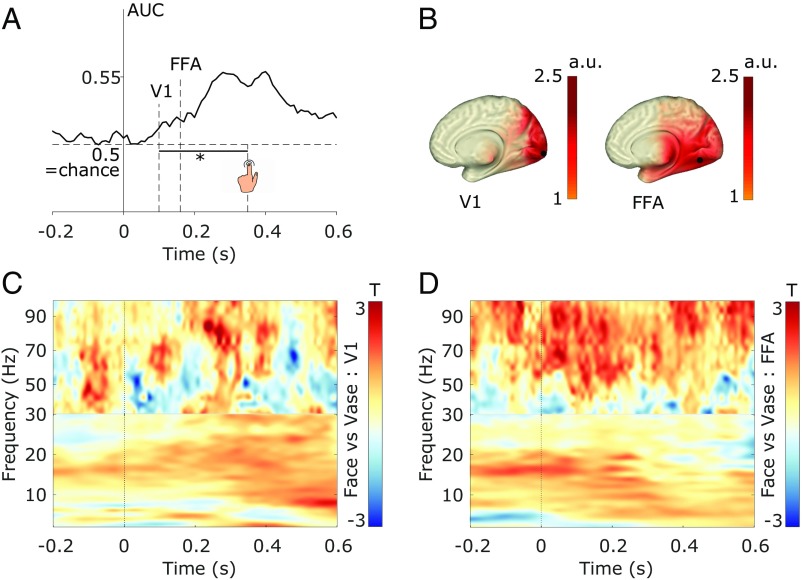
Poststimulus MEG data contains category-sensitive information with respect to the processing of the Rubin face/vase stimulus. (*A*) Temporal decoding of face vs. vase reports. * represents *P* = 0.0001 significance of decoding accuracy (*t* test vs. chance) starting at 100 ms poststimulus. (*B*) Unmasked activation maps resulting from the source reconstruction of the classifier weights (in arbitrary units [a.u.]), applying a procedure proposed by ref. 39, at different time points, suggesting temporally changing informative regions (V1 ∼100 ms and FFA ∼160 ms after stimulus onset). While the unmasked plots suggest a larger spatial spread of activity at 160 ms compared with 100 ms, applying a 95%-maximum activity threshold to extract the ROIs revealed 32 grid points (8-mm resolution) exceeding the threshold at 100 ms (V1) and 1 grid point exceeding the threshold at 160 ms (FFA). Black dots represent the grid points with maximum informative activity (V1 and FFA) in the respective time periods. (*C*) Time-frequency contrast in V1 (face vs. vase reports). Colors represent smoothed *T*-values obtained from cluster-based permutation testing of the contrast (face – vase; ns). (*D*) Time-frequency contrast in FFA (face vs. vase reports). Colors represent smoothed *T*-values obtained from cluster-based permutation testing of the contrast (face – vase; *p*_cluster_ = 0.029). Black lines surround the time-frequency gamma-range cluster that drove the significant statistical difference.

In a first step, we aimed to extract category-specific information from the recorded MEG data to see whether source localization of this information would yield the regions of interest (ROIs) found in previous work (i.e., primary visual cortex [V1] and FFA), and to later use this information to link pre- and poststimulus neural activity. For this purpose, we trained a classifier in a cross-validation approach and decoded face vs. vase reports from the MEG sensor-level data (magnetometers and gradiometers). The analysis was shifted over time on a sample-by-sample basis, yielding the temporally resolved decoding results shown in Fig. 1*A*. Decoding performance (operationalized as area under curve [AUC]) gradually increased following stimulus onset and reached a peak close to the offset of the stimulus mask, the event which prompted the response query. From there on, decoding performance gradually decreased, reaching chance level after approximately 700 ms. Decoding accuracy was significantly above chance after 100 ms (*p*_cluster_ = 9.999e-05; tested over the first 350 ms after stimulus onset to exclude the response epoch).

To find the brain regions that provided informative activity, we adapted a previously reported approach (39) which projects classifier weights from sensor to source space (for sources see Fig. 1*B*). This analysis suggested that the brain regions that provide informative activity changed over time (Fig. 1*A*). At earlier (<120 ms) time intervals, informative activity was predominantly localized in and around the right V1 (centered on Montreal Neurological Institute [MNI] coordinates: [12–88 0] mm; size: 32 grid points; grid resolution: 8 mm). In the subsequent time interval (120–200 ms), informative activity was predominantly localized in and around right FFA (MNI coordinates: [28–64 −4] mm; 1 grid point). Although informative activity also spread to left V1 and FFA, the locations of maximum activity, which we used for subsequent analyses, were located in the right hemisphere. The location, lateralization, and timing of informative neural activity corresponded well with reports on the spatiotemporal dynamics of face perception (40, 41).

Next, we performed time-frequency analysis in FFA after stimulus onset to reveal possible oscillatory differences between face and vase visual processing. We contrasted trials in which participants reported seeing faces vs. a vase and corrected for multiple time-frequency samples with a cluster-based permutation approach (42). We found that face reports showed enhanced poststimulus gamma activity (*p*_cluster_ = 0.029) compared with vase reports, consistent with the functional role of gamma activity for visual perception and specifically for face perception (43, 44). Over time, this cluster covered the entire relevant poststimulus time range (0–350 ms), and, in terms of frequencies, the cluster covered a range of 48–93 Hz (Fig. 1*D* and *SI Appendix*, Fig. S2*B*). In the lower frequencies, there were no clusters in the time-frequency maps, which contributed to the statistical effect (Fig. 1*D*). We repeated the same analysis and contrast in V1 and found no statistical differences (no time-frequency clusters; see Fig. 1*C* and *SI Appendix*, Fig. S2*A*); we ran a sensor-wise time-frequency analysis, repeated the same contrast, and found no statistical differences on the whole-brain level (no time-frequency-sensor clusters; see *SI Appendix*, Fig. S3). Finally, given that the gamma effect cluster included the time of stimulus onset, we ran control tests to ensure that there was no prestimulus gamma effect. We found that the width of the time-frequency analysis window used (300 ms) accounted for the observed temporal spread of the effect, such that narrower windows resulted in less spread (*SI Appendix*, Fig. S4). Using a time-frequency analysis window width of 100 ms revealed the gamma effect to be in the poststimulus epoch (Fig. 1*D*), and further analyses of the time course of the gamma effect confirmed this (*SI Appendix*, Fig. S5). Overall, this analysis showed that perceiving the stimulus as faces was accompanied by enhanced poststimulus gamma activity in the FFA.

**Fig. 2.**
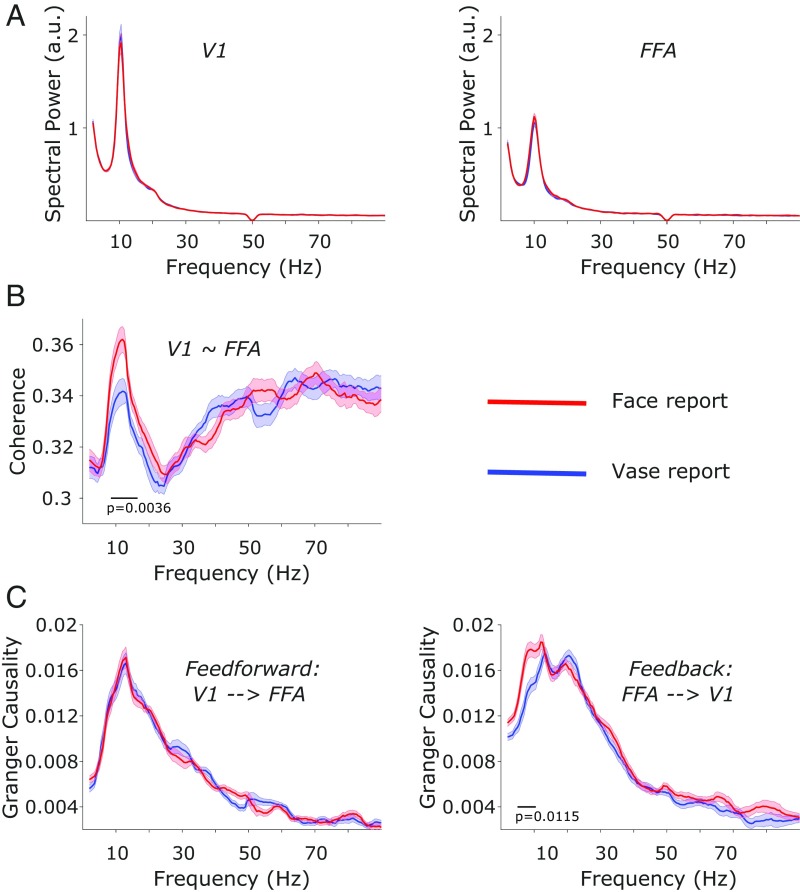
Prestimulus MEG connectivity is predictive of upcoming perceptual decisions. Shaded error regions represent SEM for within-subject designs (46). (*A*) No statistical differences in prestimulus spectral power between face and vase trials in either V1 (*Left*) or FFA (*Right*). (*B*) Compared with vase trials, face trials show increased prestimulus coherence between V1 and FFA in the alpha and beta frequency ranges. (*C*) Compared with vase trials, face trials show increased prestimulus feedback connectivity from FFA to V1 in the alpha range (*Right*) but no differences in prestimulus feedforward connectivity from V1 to FFA (*Left*) (49).

The MVPA analysis yielded favorable ROIs to test whether prestimulus connectivity dynamics between early visual regions (V1) and later category-sensitive regions (FFA) bias the report of upcoming subjective percepts (Fig. 1*A*). First, we focused on oscillatory power as an index of local excitability in these regions and tested whether excitability alone predicted the reported categories of upcoming stimuli. Oscillations reflect rhythmic changes in the activity of neural populations and thus reflect phases of high and low excitability (45). Cluster-based permutation testing revealed no statistical differences in prestimulus oscillatory power between face and vase trials, neither in V1 nor in FFA (Fig. 2*A*; shaded error regions represent SEM for within-subject designs [46]). Nevertheless, the power spectra in both conditions showed that prestimulus oscillatory activity was largely restricted to lower frequencies (5–25 Hz; Fig. 2*A*) with a clear peak in the alpha range (∼10 Hz). Since frequency-domain measures of connectivity (such as coherence or Granger causality) assume underlying oscillatory activity (i.e., oscillations with high power), we restricted statistical testing in subsequent connectivity analyses to this frequency range.

Next, we focused on prestimulus connectivity between V1 and FFA. Specifically, we hypothesized that increased prestimulus coherence between V1 and FFA would precede face reports. A cluster-based permutation test in the frequency range of 5–25 Hz revealed that prestimulus coherence between V1 and FFA was significantly greater in face vs. vase trials (*p*_cluster_ = 0.0036). This increase was most pronounced in a cluster of frequencies ranging 8.5–16.5 Hz (Fig. 2*B*). A time-resolved contrast of the coherence effect within these frequencies showed that the relative increase in face vs. vase trials was most pronounced 400–200 ms before stimulus onset (*SI Appendix*, Fig. S5). To control for spurious coherence as a result of field spread (47), which might explain the high-frequency noise in Fig. 2*B*, we repeated the coherence analysis using the imaginary part of coherency (48). We obtained qualitatively and quantitatively similar results but with far less high-frequency noise (*SI Appendix*, Fig. S6).

To further characterize the observed connectivity effect, we used Granger causality to resolve the question of whether increased connectivity before face reports represented an increased feedforward drive from V1 to FFA or an increased feedback drive from FFA to V1. We contrasted face and vase trials separately for the feedforward and feedback directions (49). The cluster-based permutation test revealed no statistical differences between face and vase reports in the prestimulus Granger causality estimates in the feedforward direction (V1 to FFA; Fig. 2 *C*, *Left*); however, for feedback connectivity, we found significantly greater prestimulus Granger causality estimates during face trials compared with vase trials (FFA to V1, *p*_cluster_ = 0.0115). This increase was most pronounced in a cluster of frequencies ranging 5–10.5 Hz (Fig. 2 *C*, *Right*). The directionalities of the Granger estimates were reversed for time-reversed data (i.e., the feedforward Granger estimates of the original data resembled the feedback Granger estimates of the time-reversed data and vice versa; see *SI Appendix*, Fig. S7), thereby confirming our results (50). Given the interindividual variability in participants’ behavioral reports (22.6–84.8% face reports), we were concerned that the Granger results might reflect some participants’ predispositions to report one or the other percept. However, we found no correlation between Granger strength and report percentages (*r* = 0.22; *P* = 0.35; see *SI Appendix*, Fig. S8), making this possibility unlikely. In sum, we show that face reports (vs. vase reports) were preceded by increased connectivity between V1 and FFA, and that this relative connectivity increase was predominantly driven by an increase in feedback connectivity (FFA to V1).

**Fig. 3.**
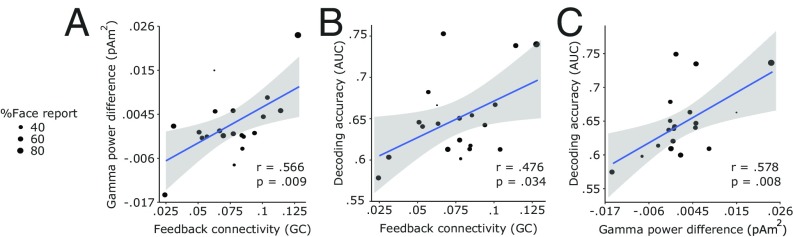
Prestimulus connectivity is correlated with poststimulus activity across participants; *r* values represent Pearson’s correlation coefficients, and shaded areas represent 95% confidence intervals. (*A*) Maximum prestimulus feedback Granger causality estimates are correlated with maximum poststimulus gamma differences (face – vase). (*B*) Maximum prestimulus feedback Granger causality estimates are correlated with maximum poststimulus decoding (AUC) scores. (*C*) Maximum poststimulus gamma differences (face – vase) are correlated with maximum poststimulus decoding (AUC) scores.

Finally, we focused on the relationship between prestimulus connectivity and poststimulus activity. We extracted for each participant the maximum decoding accuracy (AUC), FFA gamma-band effect (from the 300-ms window data), and prestimulus feedback connectivity. The maximum FFA gamma effect (maximum face – vase power over time and frequencies) and maximum decoding accuracy were correlated (*r* = 0.58; *P* = 0.008; Fig. 3*C*), despite the gamma band having been excluded from the frequency range that went into the decoder. Crucially, we found that maximum prestimulus feedback connectivity was correlated with maximum decoding accuracy (*r* = 0.48; *P* = 0.034; Fig. 3*B*) as well as maximum gamma effect (*r* = 0.57; *P* = 0.009; Fig. 3*A*). The FFA gamma-band effect from the 100-ms window data was not correlated with any of the other variables (*SI Appendix*, Fig. S9), but this short time window would not be ideal from a signal-processing perspective. In sum, we found that prestimulus feedback connectivity strength predicted not only the category of the upcoming percept but also the strength of poststimulus neural activity associated with the percept.

## Discussion

While most studies investigating the effects of prestimulus activity on perception have been concerned with determining the requisites of successfully detecting stimuli at the perceptual threshold (near-threshold paradigms; e.g., ref. 11), our main interest was with the requisites of perceiving one or another content of perception. We found that before Rubin face/vase stimulus onset, FFA was more strongly connected to V1 when faces rather than vase was subsequently reported, specifically in the feedback direction of FFA to V1. Connectivity between these two regions was concentrated in the alpha and beta frequency bands (∼5–25 Hz). Further, prestimulus feedback connectivity strength was correlated with poststimulus neural activity strength as well as decoding accuracy. Taken together, our findings suggest that fluctuations in neural activity in the absence of stimulation can bias the perceptual content of subsequently presented stimuli.

The connectivity pathway we identified, specifically the involvement of FFA, is well in line with work that has localized face responses using ambiguous stimuli (e.g., refs. 36 and 51). While this particular pathway is likely specific to face stimuli, the involvement of functionally specialized extrastriate regions in the subjective perception of ambiguous stimuli is firmly established (52). Indeed, processing semantic content typically relates to ventral stream activity, so this activity is also expected to play a crucial role in perceiving ambiguous images of semantic content, such as the Rubin vase image (53). That the connectivity pathway is in both the feedback direction and the lower frequencies is also well in line with the finding that alpha/beta oscillations subserve feedback connectivity among human (54) and monkey (55) visual cortical areas. Additionally, occipital alpha oscillations have been shown to predict the persistence of bistable perception (56), and bistable percept-dependent changes in occipital oscillatory activity have been suggested to reflect top-down modulations of V1 by extrastriate areas (57). Our findings therefore suggest that, in the absence of visual stimulation, mechanisms that mimic the known dynamics of unambiguous as well as ambiguous visual object perception are at play.

MEG studies on face perception have reported gamma responses to faces starting 100 ms after stimulus onset (e.g., refs. 58 and 59), yet we observed a statistical difference between face and vase gamma responses in FFA starting at stimulus onset. To confirm that this was genuinely a poststimulus effect, we analyzed the time-frequency data with time windows shorter than 300 ms. The analysis revealed that the early differences were strongly influenced by the width of the analysis window, as the temporal spread decreased with shorter analysis windows (*SI Appendix*, Fig. S4). Specifically, the gamma contrast from the 100-ms window data (Fig. 1*D*) illustrated that the effect was clearly poststimulus onset. The original gamma effect using the 300-ms window in FFA correlated with both the strength of prestimulus feedback connectivity and poststimulus decoding accuracy (Fig. 3). This correlation was not significant with the shorter, 100-ms window (*SI Appendix*, Fig. S9) given that shortening the time window impacts frequency resolution and can adversely affect the stability of the analysis when averaging induced responses over trials. It should be emphasized that this issue does not affect the correlation between prestimulus connectivity and poststimulus decoding effects which is central to our reasoning. Given that the prestimulus connectivity pathway was in the feedback direction from FFA to V1, one might additionally hypothesize that the strength of feedback connectivity correlates with V1 gamma activity. But V1 gamma modulations have been shown to depend on stimulus features (e.g., ref. 60), and the stimulus was unchanged throughout our experiment. Indeed, we did not observe any gamma effects in V1 (Fig. 1*C* and *SI Appendix*, Fig. S2*A*), so we could not test this hypothesis.

A recent fMRI study employing the Rubin face/vase stimulus (37) found that pre- and poststimulus neural activity was pronounced in the FFA and interpreted the observed prestimulus BOLD signal differences as differences in baseline excitability. This interpretation is consistent with a large body of work that shows that alpha-band activity in task-sensitive sensory regions, an index of neuronal excitability in those regions, predicts behavioral outcomes (8, 10, 11, 61). However, we found no differences in prestimulus alpha activity that could account for the behavioral outcome. Local excitability as indexed by alpha oscillations might therefore be behaviorally relevant in near-threshold cases but not in cases where the stimuli are suprathreshold and the task requires object perception rather than stimulus detection or discrimination. Taken together, these results show that measures of local activity paint an incomplete picture of the underlying dynamics of object perception, and that the connectivity between regions of interest must be considered for a more comprehensive account.

Given the nature of this and similar experiments (e.g., ref. 37), it is difficult to connect distinct cognitive processes to our effects with certainty. However, our findings appear to be in line with predictive processing notions that hierarchically downstream regions predict activity in upstream areas via feedback connections. The reported frequency band conforms to the assumptions and findings of this framework (62, 63). Indeed, our findings seem to add to a recent and fast-growing literature converging toward the idea that low-frequency oscillations carry top-down context (64), category information (65), anticipation (66), and expectations or predictions (67, 68). That cognitive, top-down influences might come into play leaves open the possibility that the reported connectivity effects are not strictly spontaneous and might be voluntarily driven to some extent, or that the effects reflect an expectation of the content of the upcoming stimulus. These possibilities cannot be entirely ruled out, although they are unlikely given that our design (short, temporally difficult-to-predict interstimulus intervals of 1–1.8 s) and our behavioral analysis ruled out systematic reports of one percept. Such interpretations could also be supported if we were to observe a prestimulus gamma effect, as this might indicate that the percept was fixed before stimulus onset. However, our control analyses showed that the gamma effect was in the poststimulus interval (*SI Appendix*, Figs. S4 and S5). An alternative explanation could be that stronger connectivity relates to a stronger predisposition to perceive faces. However, connectivity strength was not correlated with the percentage of face reports (*SI Appendix*, Fig. S8), making this interpretation unlikely, too.

Our results are in line with the Windows to Consciousness framework, which emphasizes the influence of preestablished connectivity patterns of relevant sensory regions to downstream processing regions on upcoming perceptual processing (11, 38). We offer a mechanistic account defined in time, space, oscillatory frequency, and directional connectivity. Our account proposes a key role of prestimulus neural fluctuations in activating connectivity pathways and biasing categorical percepts. Specifically, prestimulus feedback connectivity in the alpha range from FFA to V1 represents such a connectivity pathway that biases toward face perception in the Rubin face/vase stimulus.

## Conclusion

By recording MEG signals at high temporal resolution before and while people were exposed to an ambiguous stimulus, the Rubin face/vase illusion, we showed that the content of visual perception is critically shaped by ongoing network states—in this case, feedback alpha-band connectivity between face-sensitive FFA and the early visual cortex. Our work bridges object perception–related pre- and poststimulus effects and shows how a prestimulus network state can shape future processing in a category-sensitive brain region.

## Materials and Methods

**Fig. 4.**
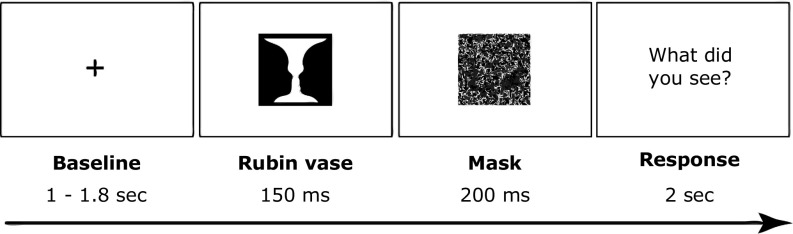
Trial structure.

20 volunteers participated in this MEG experiment. The Ethics Committee of the University of Trento approved the experimental procedure, and all participants gave written informed consent before taking part in the study. At the beginning of each trial, a fixation cross appeared at the center of the screen for 1–1.8 s. After this jittered period, the Rubin vase picture appeared at the center of the screen for 150 ms (Fig. 4). A mask stimulus then appeared for 200 ms, after which we asked participants to report whether they saw the faces or the vase. The experiment consisted of 400 trials in total.

To test for the stochastic nature of responses, we binned the data for each participant according to how many trials in a row he or she responded to with the same perceptual report. We broke this down in 11 bins with 0 to 10 repetitions, averaged the number of repetitions within each bin across participants, and then fit the averaged data to a binomial distribution across the 11 bins before calculating goodness of fit.

We performed the decoding analysis on the broadband 1–33-Hz time-domain signal. We implemented a fourfold cross-validation procedure within each subject. The analysis was shifted over time on a sample-by-sample basis. For each time point at each sensor, we *Z*-normalized the MEG data, trained a logistic regression classifier on three folds, and tested on the left-out fold. To find out which brain regions contributed most to above-chance decoding performance, we used the weights that the classifier used to separate face reports from vase reports and projected them into source space (39). Finally, we averaged the source-level weights across the intervals 50–120 and 120–200 ms and applied a 95%-maximum threshold to mask our ROIs.

We performed the poststimulus time-frequency analysis on V1, FFA, and the whole-brain average in source space. We estimated power using multitaper fast Fourier transform (FFT) with discrete prolate spheroidal sequences (DPSS) tapers (69). We calculated power, coherence, and nonparametric Granger causality (70) in the prestimulus period between FFA and V1 in source space. We used multitaper frequency transformation to obtain Fourier coefficients in the prestimulus period (−1 to 0 s), after which we extracted power and computed coherence and bivariate Granger causality. This gave us separate estimates of connection strengths from FFA to V1 (feedback) and vice versa (feedforward). We repeated the same Granger causality analysis on time-reversed data, expecting reversals in the directionalities of the estimates to rule out spurious connectivity results (50).

We tested decoding performance against chance level (50%) using one-sided dependent-sample *t* tests. For all remaining statistical analyses, we used nonparametric cluster permutation tests (42). We used two-sided *t* tests for the poststimulus time-frequency contrasts and prestimulus power contrasts, and one-sided *t* tests for the coherence and feedforward and feedback connectivity contrasts, as we had hypothesized greater values of these measures on face trials compared with vase trials. We restricted the statistical testing window of coherence and Granger to the frequency window 5–25 Hz.

More details about the participants, experimental procedure, MEG data acquisition, MEG preprocessing and source projection, and all reported analyses are available in the *SI Appendix*, *Materials and Methods* section.

## Supplementary Material

Supplementary File
