# Supplementary material for: Prestimulus feedback connectivity biases the content of visual experiences
Source: Proc Natl Acad Sci U S A. 2019 Jul 22;116(32):16056–61. doi: 10.1073/pnas.1817317116 (PMC6689959; doi:10.1073/pnas.1817317116)
Supplement: Supplementary File [file pnas.1817317116.sapp.pdf]

PNAS
www.pnas.org

Supplementary Information for

Pre-stimulus feedback connectivity biases the content of visual experiences

Elie Rassi, Andreas Wutz, Nadia Müller-Voggel, & Nathan Weisz

Elie Rassi
Email: elie.elrassi@sbg.ac.at

This PDF file includes:

Supplementary Information Materials and Methods
Figs. S1 to S5
References for SI reference citations

www.pnas.org/cgi/doi/10.1073/pnas.1817317116

Supplementary Information Materials and Methods

Participants

20 right-handed volunteers with normal or corrected-to-normal vision participated in this experiment (9 m/11 f, mean age 25.3). Before the experiment, participants filled a form declaring they had no metal objects on their bodies. During the course of the experiment, participants wore non-magnetic clothes.

Experimental Procedure

After we placed the Head Position Indicator (HPI) coils on the participants' head, the experiment proper began. Participants sat upright in the MEG system. We instructed them to keep fixation and to avoid eye blinks and movements as best as possible during the experiment. In between the blocks participants took a short break but remained seated in the MEG system. We displayed the visual stimuli via a video projector outside of the MEG chamber. It projected to a back-projection screen in the MEG chamber. We monitored participants via video camera as they performed the experimental task. At the beginning of each trial, a fixation cross would appear at the centre of the screen for 1 to 1.8 s. After this jittered period, the Rubin vase picture would appear at the centre of the screen for 150 ms (see main text **Fig. 4** for trial example). We presented half of the participants with the original Rubin vase picture (black background) and the other half with a colour inverted version (white background). We did this to ensure that the luminance of the picture did not bias the dominant percept, and post-hoc group analysis revealed no differences between the types of background on the measures reported. After the Rubin vase picture presentation, a mask stimulus would appear for 200 msec. We created this mask by randomly scrambling blocks of pixels of the face-vase picture. After the mask presentation, we asked participants to report whether they saw the face or the vase. The response window was 2 s; if participants did not respond within that window, the next trial would start. Participants responded with the index and middle finger of the left or right hand. We counter-balanced the response hands across subjects. The experiment consisted of 400 trials in total, broken into 4 blocks of 100 trials. We controlled the visual stimulus presentation

with Matlab (1) and the Psychophysics Toolbox (2), and corrected the timings using a photo diode. The procedure of the experiment is illustrated in the main text **Fig. 4**.

Behavioural Analysis

We collected behavioural reports after the end of each trial, giving us 400 responses. To test for the stochastic nature of the response we used curve-fitting procedures from the curve-fitting toolbox in Matlab. Specifically, for each participant we binned the data according to how many trials in a row they responded with the same perceptual report. We broke this down in 11 bins with 0 repetitions to 10 repetitions, averaged the number of repetitions within each bin across participants, and then fit the averaged data to a binomial distribution generated in Matlab across the 11 bins before calculating goodness-of-fit.

MEG Data Acquisition

We carried out the MEG recordings using a 306-channel whole-head VectorView MEG system (Elekta-Neuromag, Ltd., Helsinki, Finland, 204 gradio- and 102 magnetometers) installed in a magnetically shielded chamber (AK3b, Vakuumschmelze Hanau, Germany), with signals recorded at 1000 Hz sample rate. Hardware filters were adjusted to band-pass the MEG signal in the frequency range of 0.01 Hz to 330 Hz. Prior to the recording, we recorded points on the participant's head using a digitizer (Polhemus, VT, USA). These points included the 5 HPI coils, the three fiducials (nasion, left and right pre-auricular points), and over 200 additional points covering the head as evenly as possible. We used the HPI coils to monitor head position during the experiment.

MEG Preprocessing and Source Projection

We pre-processed the data using the Fieldtrip toolbox (3). From the raw continuous data, we extracted epochs of 4 seconds lasting from 2.5 seconds before onset of the picture to 1.5 seconds after onset of the picture. This resulted in 400 trials per participant. We applied a high-pass filter on this epoched data at 1 Hz (IIR Butterworth 6-order two-pass filter with 36 dB/oct roll-off), followed by a band-stop filter of 49 – 51Hz to remove power line noise. We then down-sampled the data to 400 Hz. We visually inspected the trials for strong non-physiological artefacts (e.g. channel jumps) and rejected the contaminated trials before computing ICA. We removed components representing typical physiological artefacts (e.g. blinks, ECG) and

reconstructed the cleaned raw data. We finally removed the remaining noisy trials by visual inspection. For each participant, we then assigned the trials to the 2 conditions according to the participants' response. Although there was almost equal incidence of face and vase reports to start with, the balance of trial numbers changed after artefact rejection. To ensure a similar signal-to-noise-ratio across conditions, we equalized the trial numbers of face and vase reports by random omission (percentage of trials left in the analysis: M = 79.32%, SD = 15.12%).

We projected the data to source space by applying LCMV (linear constrained mean variance) beamformer filters to the sensor level data (4). We created anatomically realistic headmodels (5) using participants' individual structural MRI and the Polhemus digitized scalp shape. For three participants for which we could not obtain an individual MRI, we used a template MRI which was morphed to fit the individuals head shape using an affine transformation. We calculated a three-dimensional source grid (resolution: 8 mm) covering an entire MNI standard brain volume. For each of these points, we computed an LCMV filter using the individual leadfield and the data covariance matrix (estimated separately for the focus of analysis; see below). We used these spatial filters to then project classifier weights into source space and compute oscillatory and connectivity measures for distinct ROIs (see next sections).

Multivariate Pattern Analysis (MVPA)

We resampled the MEG data to 100 Hz to speed up the MVPA computations (6, 7) using an algorithm that first applies a low-pass filter at one third of the resampling frequency. So, we performed the decoding analysis on the broadband 1-33 Hz time-domain signal. We used MNE Python (8) which uses Scikit-learn (9, 10) for the decoding and implemented a 4-fold cross-validation procedure within each subject. The analysis was shifted over time on a sample-by-sample basis. For each time-point at each sensor, we Z-normalized the MEG data, trained a Logistic Regression classifier on three folds, and tested on the left-out fold. We operationalized the decoding performance as Area Under the Curve (AUC).

To find out which brain regions contributed to above chance decoding performance the most, we used the classifier weights that the classifier used to separate face from vase reports. To obtain interpretable sensor-level topographies, we multiplied the classifier weights by the data covariance in a first step (11). Then we applied LCMV beamformer filters (using a -.3 to .35 s window to calculate the covariance matrix) to project the weights into source space. At the source level, we abolished polarity differences across voxels by taking the absolute values. This

approach is near-identical to the "informative activity" procedure reported in a recent study (12). Finally, we averaged the source-level weights across the intervals 50 to 120 ms and 120 to 200 ms and applied a 95%-max threshold to mask our ROIs. This resulted in a V1 ROI centred around MNI coordinates [12 -88 0] mm with a size of 32 grid points and a FFA ROI centred around MNI coordinates [28 -64 -4] mm with a size of 1 grid point (8mm grid resolution).

Analysis of Post-stimulus Oscillatory Activity

The MVPA provided a clear ROI in right ventromedial temporal cortex corresponding to Fusiform Face Area (FFA) at expected latencies (around 160 ms). We performed time-frequency analysis specifically for this ROI by using the single-trial source-projected time series (using the full epoch length for calculation of the covariance matrix). We estimated power using multitaper Fast Fourier Transform (FFT) with discrete prolate spheroidal sequences (dpss) (13), with separate window lengths (.5 s for low frequencies [2-30 Hz in 1 Hz steps] and .3 s for high frequencies [33-99 Hz in 3 Hz steps]). We adapted smoothing to the specific frequencies for which we estimated power (linearly increasing from 2 to 6 Hz for low frequencies and set to +/- 20% of the center frequencies for high frequencies). As a control analysis, we repeated the same procedure for high frequencies using window lengths of 0.2 s and 0.1 s.

Analysis of Pre-stimulus Power, Coherence, and Granger Causality

In addition to the face-sensitive region FFA, the classifier weights source analysis implied the involvement of V1 at earlier time points. We calculated power, coherence, imaginary part of coherency, and Nonparametric Granger causality (14) in the pre-stimulus period between FFA and V1 in source space (using the full epoch length to calculate the covariance matrix). We used multi-taper frequency transformation with a spectral smoothing of 2 Hz to get Fourier coefficients in the pre-stimulus period (-1 to 0 s), after which we extracted power and computed coherence and bivariate Granger causality. This gave us separate estimates of connection strengths from FFA to V1 ("feedback") and vice versa ("feedforward"). We repeated the same Granger causality analysis on time-reversed data, expecting reversals in the directionalities of the estimates to rule out spurious connectivity results (15). That is, we expected the feedforward Granger estimates of the original data to differ from those of the time-reversed data, and to instead resemble the feedback estimates of the original data, and vice versa.
We averaged all results over all grid points within the V1 ROI.

As a control analysis, we calculated time- and frequency-resolved coherence based on the time-frequency data obtained as described in the previous section, and averaged across the frequencies between 50 and 90 Hz to obtain a coherence time-course.

Statistical Analysis of MEG Data

For the MVPA analysis, we tested decoding performance against chance level (50%) using a dependent-samples T-test. Since we were interested in periods in which the classifier performs above chance, we used a one-sided T-test. For all remaining statistical analyses, we used nonparametric cluster permutation tests (16), comparing a selected test statistic against a distribution obtained from 10000 permutations. We set thresholds for forming clusters at $p < .05$ and considered an effect significant if its probability with respect to the nonparametric distribution was $p < .05$. For the post-stimulus time-frequency contrast in FFA, we tested power in the face-vase contrast in the window of 0 to .35 s, separately for the low and high frequencies, using 2-sided dependent-samples T-tests, and without applying a baseline correction. We did the same for pre-stimulus power, except that this estimate was not time-resolved. We tested coherence and feedforward and feedback connectivity with 1-sided dependent-samples T-tests as we had hypothesized greater values of these measures on face trials compared to vase trials. We restricted the statistical testing window of coherence and Granger to the frequency window 5-25 Hz, which covers the visible peaks in the grand-averaged power and coherence spectra.

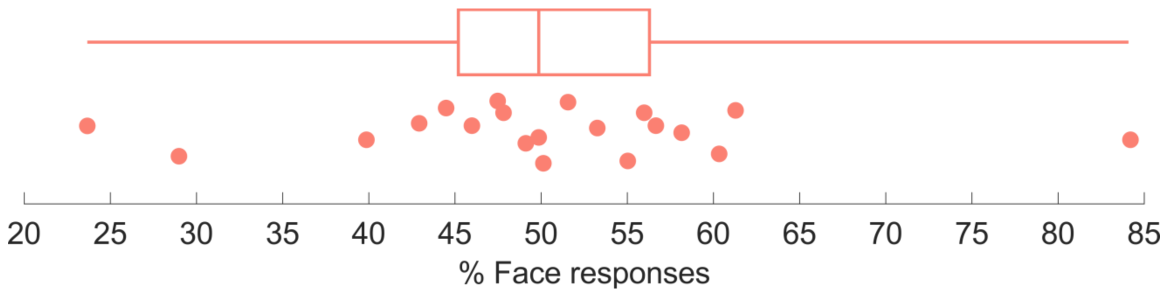

Figure S1: Behavioral responses (percentage of times each participant reported seeing faces). Dots represent individual participants and boxplot represents median and quartiles.

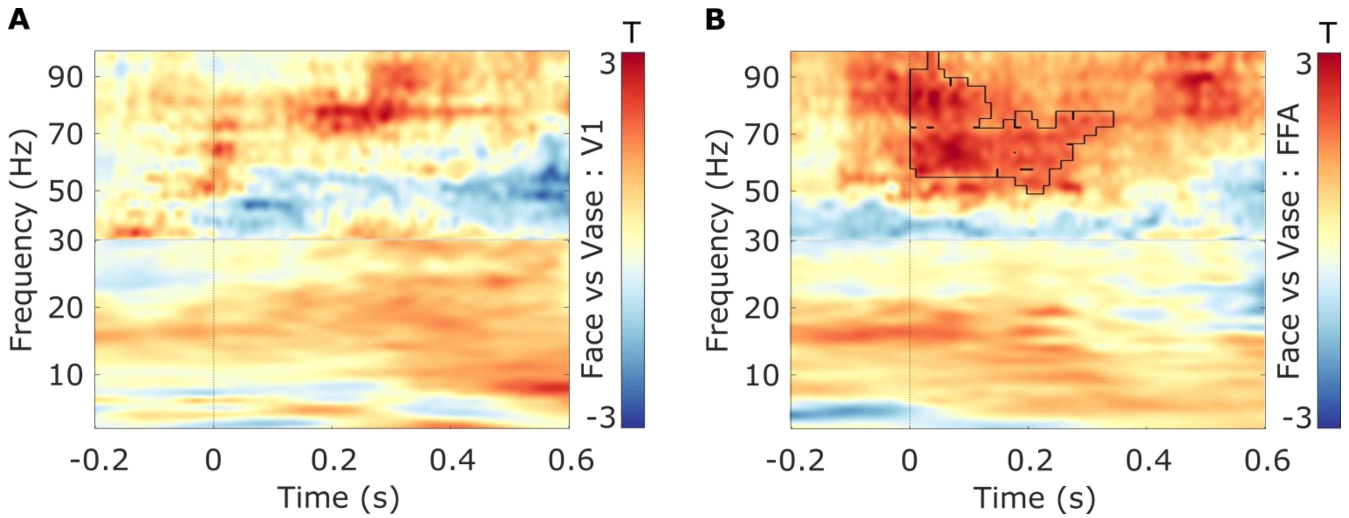

Figure S2: A) Time-frequency contrast in V1 (face vs vase reports) using 300 ms analysis window for the frequencies 33 – 99 Hz. Colors represent smoothed T-values obtained from cluster-based permutation testing of the contrast (face – vase; *ns*). B) Time-frequency contrast in FFA (face vs vase reports) using 300 ms analysis window for the frequencies 33 – 99 Hz. Colors represent smoothed T-values obtained from cluster-based permutation testing of the contrast (face – vase; $p_{cluster}$ = .029). Black lines surround the time-frequency gamma-range cluster that drove the significant statistical difference.

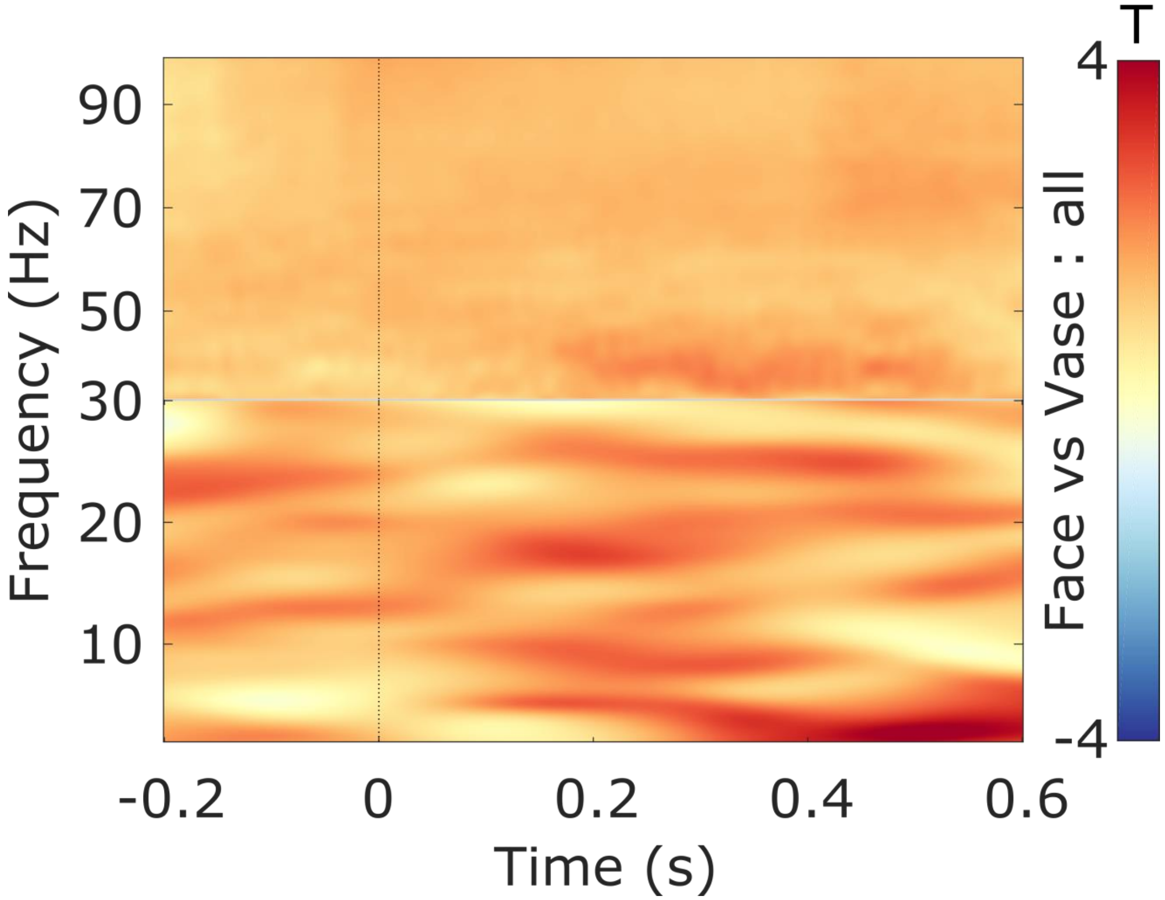

Figure S3: Sensor-wise time-frequency contrasts (face vs vase reports) with cluster-based permutation testing revealed no time-frequency-sensor clusters. Colors represent smoothed T-values of the contrast, averaged across all sensors (204 gradiometers and 102 magnetometers).

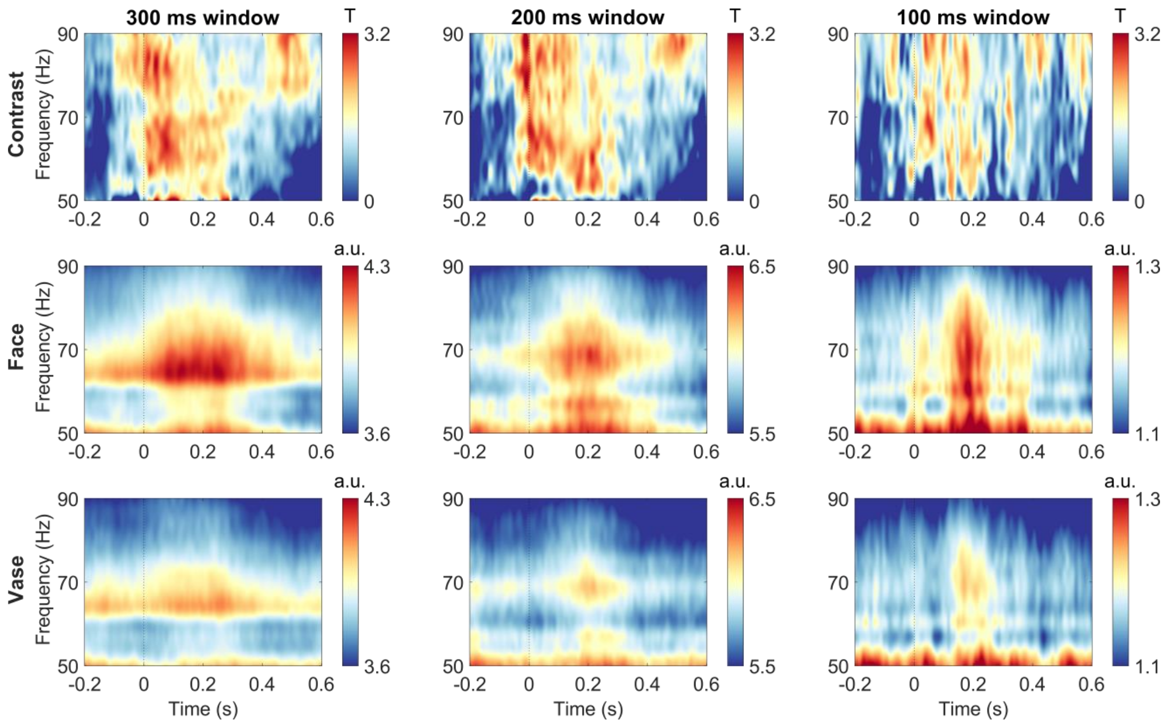

Figure S4: The apparently early onset of the gamma effect in FFA is a consequence of the width of the time window used for the time-frequency analysis, such that the apparent onset of the effect precedes the reported 40 ms peak by a period approximately equivalent to half of the analysis window width. Top-left: with an analysis window of 300 ms, the apparent onset of the effect is -100 ms, or around 150 ms before the reported 40 ms peak. Top-middle: with an analysis window of 200 ms, the apparent onset of the effect is -50 ms, or around 100 ms before the reported 40 ms peak. Top-right: with an analysis window of 100 ms, the apparent onset of the effect is 0 ms, or around 50 ms before the reported 40 ms peak. Middle row: Grand-averaged time-frequency plots of FFA gamma activity on face trials. Bottom row: Grand-averaged time-frequency plots of FFA gamma activity on vase trials. Top row colors represent smoothed T-values obtained from cluster-based permutation testing of the time-frequency power contrast (face – vase). Middle and bottom row colors represent smoothed time-frequency power values in arbitrary units.

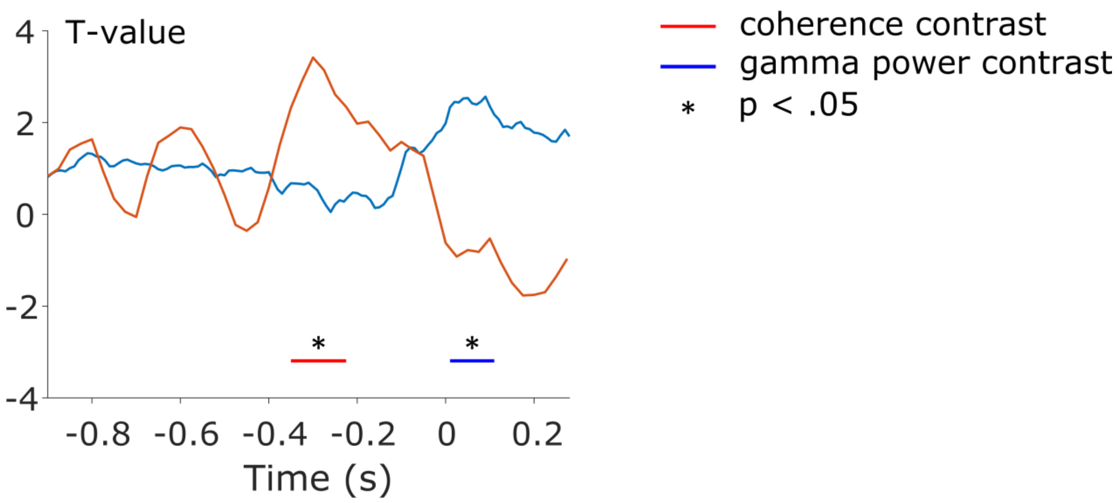

Figure S5: The time-courses of the connectivity effect and the gamma effect show that the pre-stimulus connectivity effect precedes the post-stimulus gamma effect by around 350 ms. The time-courses represent T-values obtained from cluster-based permutation testing of the face vs vase effects. The red time-course represents T-values obtained from cluster-based permutation testing of the face vs vase coherence contrast, averaged over the frequencies 5 – 25 Hz, and clustering over the time dimension. The blue time-course represents T-values obtained from cluster-based permutation testing of the face vs vase time-frequency power contrast, averaged over the gamma frequencies 50 – 90 Hz, and clustering over the time dimension from -1 s to 0.2 s. The thick horizontal red and blue lines plotted below the time-courses indicate the time periods which drove the significant statistical differences ($p_{cluster} < .05$).

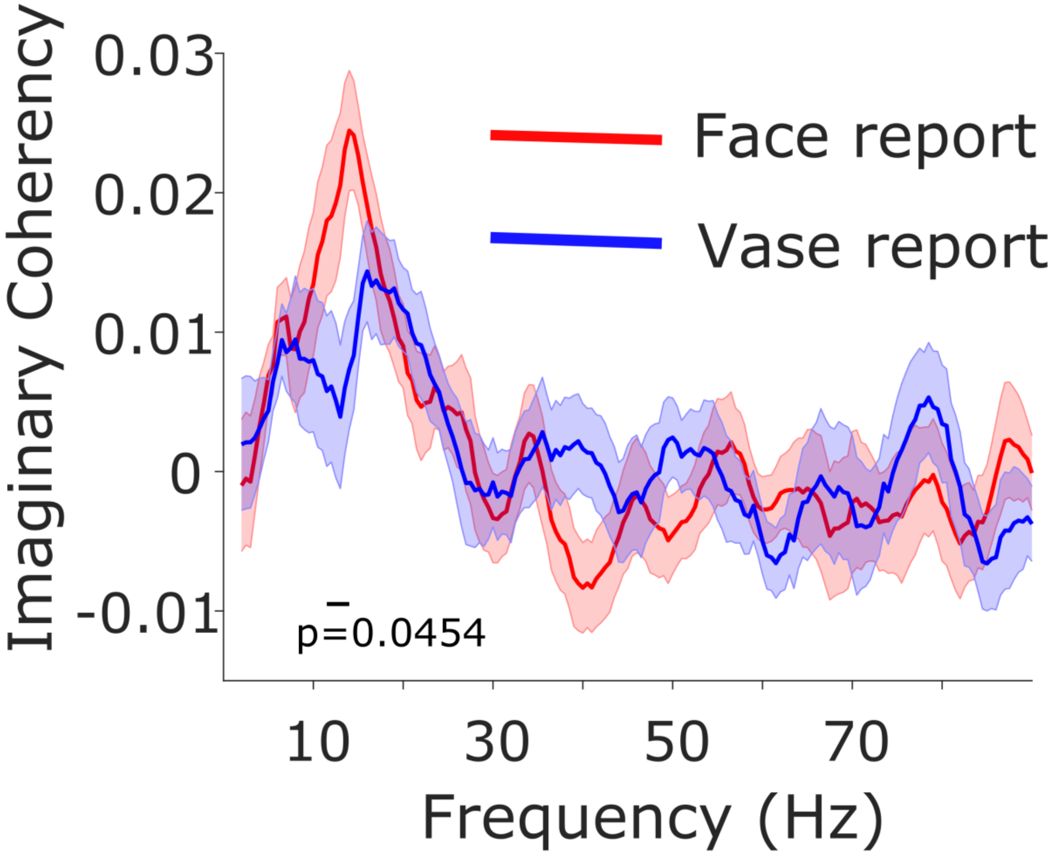

Figure S6: Pre-stimulus contrast (face vs vase) of imaginary part of coherency produces the same result as the coherence contrast, but with less high frequency noise. Red line is imaginary coherency on face trials and blue line on vase trials. Shaded error regions represent the standard error of the mean for within-subject designs (17). Compared to vase trials, face trials show increased pre-stimulus imaginary coherency between V1 and FF1 in the alpha/beta frequency range.

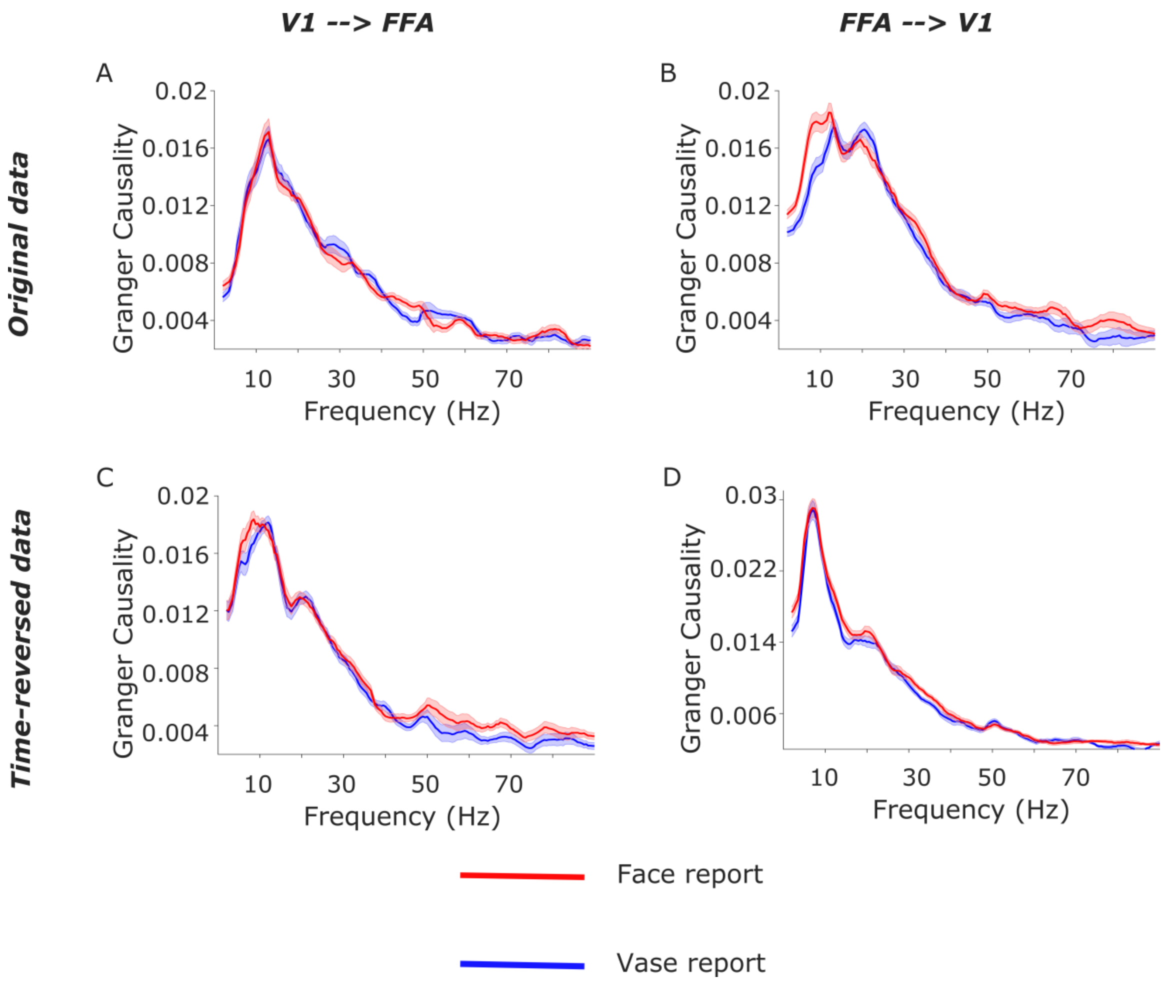

Figure S7: Granger Causality estimates on time-reversed data reveal an expected reversal in the directionality of results compared to original data, thereby increasing confidence that the observed results are not a product of co-varying noisy sources. Shaded error regions represent the standard error of the mean for within-subject designs (17). A) Feedforward (V1 -> FFA) Granger causality estimates on original data (a copy of **Fig. 2C**, left). B) Feedback (FFA -> V1) Granger causality estimates on original data (a copy of **Fig. 2C**, right). C) V1 -> FFA Granger Causality estimates on time-reversed data resemble FFA -> V1 in original data (B), but not V1 -> FFA in original data (A). D) FFA -> V1 Granger Causality estimates on time-reversed data resemble V1 -> FFA connectivity in original data (A), but not FFA -> V1 in original data (B).

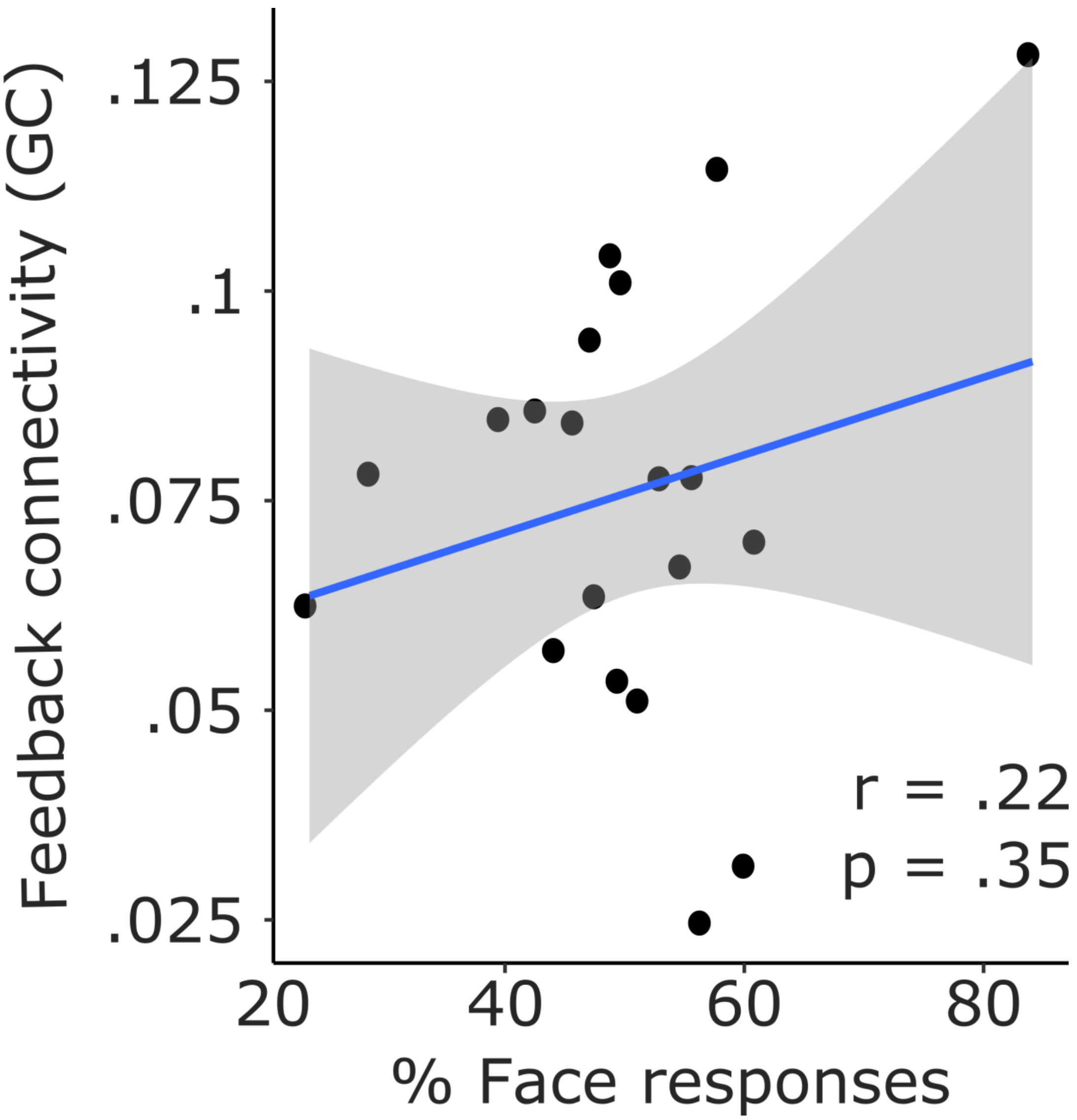

Figure S8: No correlation between feedback Granger Causality and percent face reports across participants. r value represents Pearson's correlation coefficients. Shaded area represents 95% confidence intervals.

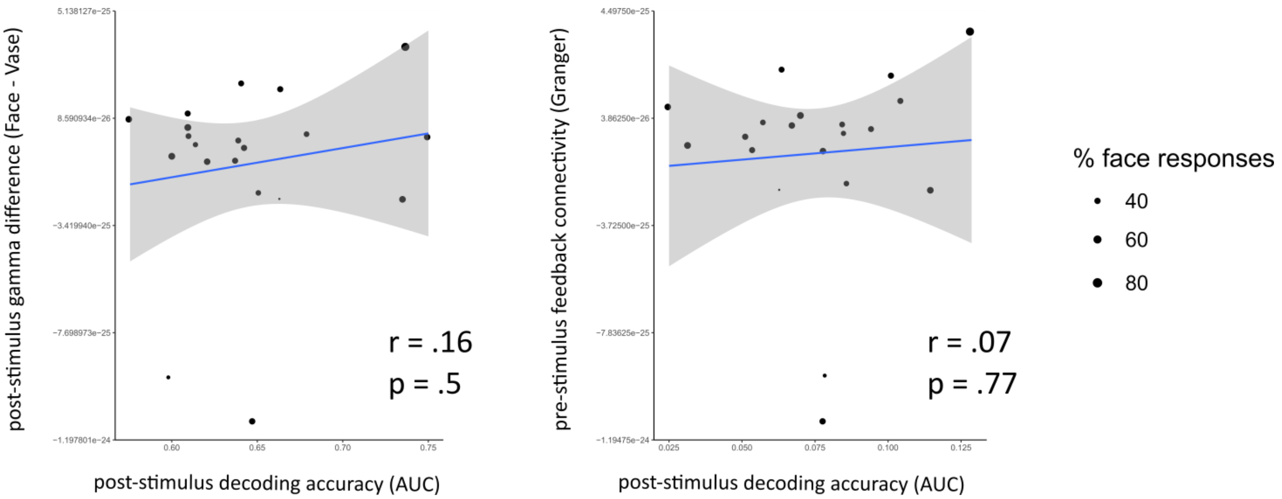

Figure S9: No correlation between the maximum gamma effect (obtained from the 100 ms analysis window data) and either pre-stimulus feedback Granger or post-stimulus decoding accuracy. r value represents Pearson's correlation coefficient. Shaded area represents 95% confidence intervals.

References

1. M. U. S. Guide, The mathworks. *Inc., Natick, MA* **5**, 333 (1998).
2. M. Kleiner, D. Brainard, & D. Pelli, What's new in Psychtoolbox-3? *Perception* **36**, 14-14 (2007).
3. R. Oostenveld, P. Fries, E. Maris, & J. M. Schoffelen, FieldTrip: Open source software for advanced analysis of MEG, EEG, and invasive electrophysiological data. *Comput Intell Neurosci* **2011**, 156869 (2011).
4. B. D. Van Veen, W. van Drongelen, M. Yuchtman, & A. Suzuki, Localization of brain electrical activity via linearly constrained minimum variance spatial filtering. *IEEE Trans Biomed Eng* **44**, 867-880 (1997).
5. G. Nolte, The magnetic lead field theorem in the quasi-static approximation and its use for magnetoencephalography forward calculation in realistic volume conductors. *Phys Med Biol* **48**, 3637-3652 (2003).
6. D. Kaiser, N. N. Oosterhof, & M. V. Peelen, The Neural Dynamics of Attentional Selection in Natural Scenes. *J Neurosci* **36**, 10522-10528 (2016).
7. J. R. King, N. Pescetelli, & S. Dehaene, Brain Mechanisms Underlying the Brief Maintenance of Seen and Unseen Sensory Information. *Neuron* **92**, 1122-1134 (2016).
8. A. Gramfort, M. Luessi, E. Larson, D. A. Engemann, D. Strohmeier, C. Brodbeck, L. Parkkonen, & M. S. Hamalainen, MNE software for processing MEG and EEG data. *Neuroimage* **86**, 446-460 (2014).
9. A. Abraham, F. Pedregosa, M. Eickenberg, P. Gervais, A. Mueller, J. Kossaifi, A. Gramfort, B. Thirion, & G. Varoquaux, Machine learning for neuroimaging with scikit-learn. *Front Neuroinform* **8**, 14 (2014).
10. F. Pedregosa, G. Varoquaux, A. Gramfort, V. Michel, B. Thirion, O. Grisel, M. Blondel, P. Prettenhofer, R. Weiss, V. Dubourg, J. Vanderplas, A. Passos, D. Cournapeau, M. Brucher, M. Perrot, & E. Duchesnay, Scikit-learn: Machine Learning in Python. *J Mach Learn Res* **12**, 2825-2830 (2011).
11. S. Haufe, F. Meinecke, K. Gorgen, S. Dahne, J. D. Haynes, B. Blankertz, & F. Biessmann, On the interpretation of weight vectors of linear models in multivariate neuroimaging. *Neuroimage* **87**, 96-110 (2014).
12. S. Marti & S. Dehaene, Discrete and continuous mechanisms of temporal selection in rapid visual streams. *Nat Commun* **8**, 1955 (2017).
13. P. P. Mitra & B. Pesaran, Analysis of dynamic brain imaging data. *Biophys J* **76**, 691-708 (1999).
14. M. Dhamala, G. Rangarajan, & M. Ding, Analyzing information flow in brain networks with nonparametric Granger causality. *Neuroimage* **41**, 354-362 (2008).
15. S. Haufe, V. V. Nikulin, K. R. Muller, & G. Nolte, A critical assessment of connectivity measures for EEG data: a simulation study. *Neuroimage* **64**, 120-133 (2013).
16. E. Maris & R. Oostenveld, Nonparametric statistical testing of EEG- and MEG-data. *J Neurosci Methods* **164**, 177-190 (2007).

17. R. D. Morey, Confidence intervals from normalized data: A correction to Cousineau (2005). *reason* **4**, 61-64 (2008).
